# DelIrium VULnerability in GEriatrics (DIVULGE) study: a protocol for a prospective observational study of electroencephalogram associations with incident postoperative delirium

**DOI:** 10.1136/bmjno-2021-000199

**Published:** 2021-12-06

**Authors:** Monique S Boord, Daniel H J Davis, Peter J Psaltis, Scott W Coussens, Daniel Feuerriegel, Marta I Garrido, Alice Bourke, Hannah A D Keage

**Affiliations:** 1 Cognitive Ageing and Impairment Neurosciences Laboratory, Justice and Society, University of South Australia, Adelaide, South Australia, Australia; 2 MRC Unit for Lifelong Health and Ageing, UCL, London, UK; 3 Vascular Research Centre, Heart and Vascular Program, Lifelong Health Theme, South Australian Health and Medical Research Institute, Adelaide, South Australia, Australia; 4 Adelaide Medical School, University of Adelaide, Adelaide, South Australia, Australia; 5 Department of Cardiology, Royal Adelaide Hospital, Central Adelaide Local Health Network, Adelaide, South Australia, Australia; 6 Melbourne School of Psychological Sciences, University of Melbourne, Melbourne, Victoria, Australia; 7 Aged Care, Rehabilitation and Palliative Care (Medical), Northern Adelaide Local Health Network, Adelaide, South Australia, Australia

**Keywords:** event-related potentials, cognitive electrophysiology, EEG, geriatrics, neurophysiology

## Abstract

**Introduction:**

Delirium is a neurocognitive disorder common in older adults in acute care settings. Those who develop delirium are at an increased risk of dementia, cognitive decline and death. Electroencephalography (EEG) during delirium in older adults is characterised by slowing and reduced functional connectivity, but markers of vulnerability are poorly described. We aim to identify EEG spectral power and event-related potential (ERP) markers of incident delirium in older adults to understand neural mechanisms of delirium vulnerability. Characterising delirium vulnerability will provide substantial theoretical advances and outcomes have the potential to be translated into delirium risk assessment tools.

**Methods and analysis:**

We will record EEG in 90 participants over 65 years of age prior to elective coronary artery bypass grafting (CABG) or transcatheter aortic valve implantation (TAVI). We will record 4-minutes of resting state (eyes open and eyes closed) and a 5-minute frequency auditory oddball paradigm. Outcome measures will include frequency band power, 1/f offset and slope, and ERP amplitude measures. Participants will undergo cognitive and EEG testing before their elective procedures and daily postoperative delirium assessments. Group allocation will be done retrospectively by linking preoperative EEG data according to postoperative delirium status (presence, severity, duration and subtype).

**Ethics and dissemination:**

This study is approved by the Human Research Ethics Committee of the Royal Adelaide Hospital, Central Adelaide Local Health Network and the University of South Australia Human Ethics Committee. Findings will be disseminated through peer-reviewed journal articles and presentations at national and international conferences.

**Trial registration number:**

ACTRN12618001114235 and ACTRN12618000799257.

**Strengths and limitations of this study**

- Our prospective design measuring electroencephalography (EEG) before delirium will allow characterisation of neural mechanisms associated with delirium vulnerability.
- We will use state-of-the-art EEG analysis and visualisation methods to elucidate neural mechanisms.
- We will extend on previous studies by assessing at the delirium subtype level and by measuring event-related potentials.
- This study is limited geographically to Adelaide, South Australia, and is limited by our inability to balance subtype sample sizes.

## Introduction

Delirium is a serious neurocognitive disorder that is seen in 20%–40% of older adults undergoing surgery.^1–3^ It is characterised as a fluctuating disturbance in attention and awareness over a short period (hours–days) accompanied by a disturbance in cognition.^1–3^ Delirium is associated with many adverse outcomes in older adults, including a ninefold increased risk of incident dementia,^4^ 41% increased likelihood of long-term cognitive impairment^5^ and a three-fold increased risk of mortality at 1 year.^6^ Delirium is a considerable financial burden worldwide, costing the Australian healthcare system AUD$8.8 billion in the 2016/2017 financial year^7^, and between US$143 and US$152 billion in the USA as reported in 2011.^8^

Delirium subtypes include hypoactive, hyperactive and mixed.^9^ Hypoactive delirium is characterised by decreased activity and amount or speed of speech, along with reduced awareness, while hyperactive delirium presents with increased activity, agitation and hallucinations.^10 11^ Displaying features of both hypoactive and hyperactive delirium characterises mixed delirium.^10^ Hypoactive delirium, as compared with other motor subtypes, has generally been associated with increased mortality and worse long-term cognition.^12^ Along with different prognoses, each subtype demands different hospital care.^13^

Delirium is conceptualised as a disorder of brain disintegration,^14–16^ and delirium vulnerability (ie, high risk of incident delirium) is thought to be driven by reduced baseline functional connectivity.^17^ There has been a recent call for subtype features to be assessed, with implications for better understanding the underlying neurobiology of delirium.^1^

Electroencephalography (EEG) is a portable and non-invasive functional neuroimaging technique, which can be used during rest and cognitive tasks. It measures summed postsynaptic excitatory and inhibitory potentials from the scalp with excellent temporal resolution.^18 19^ Spectral analysis is a standard measure reflecting the amount of periodic activity (sometimes termed oscillatory) in predefined frequency bands, for example, delta (1–4 Hz), theta (4–8 Hz), alpha (8–13 Hz), beta (13–30 Hz) and gamma (30–100 Hz).^14^ Event-related potentials (ERPs) can be extracted from EEG data and reflect deflections in voltage time-locked to events.^20^ ERPs provide dynamic information about sensory and cognitive processes and can index activity before, during and after the onset of a stimulus on a millisecond-by-millisecond basis.^21^ EEG has successfully provided neural markers of numerous clinical disorders, including schizophrenia,^22–24^ coma,^25 26^ psychosis^27 28^ and depression,^29 30^ and is a promising approach to capture delirium vulnerability.^31^

Our recent systematic review summarised EEG associations with delirium relative to time, that is, before (vulnerability for delirium), during, and after delirium.^31^ These time-points are relevant as the EEG can be affected by surgical and situational factors. For example, EEG recorded during surgery is known to be affected by anaesthesia and other events including hypothermia.^32^ In our review, EEG at the time of a delirium episode was consistently associated with slowing, predominantly characterised by higher delta and theta power, along with lower alpha power.^31^ Only two studies measured EEG before both the precipitant and the manifestation of delirium, a time-point unaffected by surgical factors, with neither reporting significant differences in relative delta power and EEG hemispheric symmetry between those who did and did not go on to develop delirium.^31^

A recent prospective study collected preoperative and postoperative EEG, along with preoperative MRI^33^. They reported that those who went on to develop delirium had higher preoperative alpha power, increased alpha-band functional connectivity and increased radial diffusivity.^33^ Increased functional connectivity was interpreted as a compensatory mechanism for maintaining cognitive function in the presence of underlying structural degeneration, which was then overwhelmed by mechanisms of delirium.^33^ The EEG recording consisted of 15 min of eyes-closed resting state, and the possibility of periods of sleep cannot be excluded.^34^ We consider it essential to assess both eyes-open and eyes-closed states, given the arousal systems are key in delirium neurobiology, and to control for baseline cognitive function, given cognitive impairment is a known delirium risk factor.^35–39^

EEG delirium markers are the result of underlying neurobiological processes. Multiple neurotransmitter systems (and their interactions) are implicated in the development of delirium, including acetylcholine, gamma-aminobutyric acid (GABA), norepinephrine, serotonin and dopamine.^15 40 41^ The role of acetylcholine is heavily involved in two key features of delirium: attention^42^ and arousal.^40^ Acetylcholine abnormalities can disrupt sensory input, giving rise to delirium symptoms, including inattention, disorganised thinking and perceptual disturbances.^40^ Increases in dopamine may lead to hyperactive symptoms, including hallucinations, agitation and irritability, due to the inhibition of the ability for catechol-O-methyl transferase to break down dopamine in the prefrontal cortex.^43 44^ GABAergic medications, including benzodiazepines, are a precipitant of delirium.^17^ Neurotransmitter levels correlate with EEG indices, for example, early ERP components appear to be modulated by cholinergic medication, and low levels of cholinergic acetyltransferase have been associated with increased delta power.^45 46^ GABAergic, glutamatergic and cholinergic neurotransmission are important for predictive attentional processes, such as those indexed by the mismatch negativity (MMN) ERP component during the auditory oddball paradigm.^42 47^

Delirium is a whole-brain disorder, representing an extensive failure of normal brain function. This failure is undoubtedly the result of widespread network disintegration with disturbances within and between arousal systems and cognitive networks.^37 48^ EEG spectral profiles and patterns of ERP componentry between delirium subtypes have not been investigated. Increases in neurotransmitters such as norepinephrine have been thought to contribute to symptoms characteristic of hyperactive and mixed delirium such as hypervigilance, while changes in GABA and serotonin may potentially be predominantly involved in hypoactive delirium.^41 44^ In other disorders where hypervigilance is a defining characteristic (eg, post-traumatic stress disorder), EEG changes have been observed, including increased MMN amplitude.^49^ In contrast, states of hypo-arousal characterising disorders such as attention deficit hyperactivity disorder, are associated with EEG slowing and attenuated ERP components.^50–52^ It is not yet clear whether EEG spectral profiles and patterns of ERP components differ between delirium subtypes. Still, given the interactions between neurotransmission, EEG changes and behavioural symptoms, we expect that delirium subtypes (hyperactive, hypoactive and mixed) and no delirium will relate differently to EEG power and ERP indices.

The DIVULGE study aims to characterise the neural mechanisms underlying vulnerability to delirium and its subtypes using EEG and ERPs. We will employ a prospective observational design, measuring EEG in older adults prior to elective cardiac surgery that may precipitate delirium. We will use state-of-the-art EEG data processing and visualisation methods to assess differences in EEG power along with ERP amplitudes and latencies between those who do and do not go on to develop delirium. We will also determine the effects of delirium subtype, severity and duration. We will extract EEG data in the form of periodic and aperiodic power spectra from resting data (eyes open and eyes closed) and ERP component amplitude and latencies from an auditory oddball paradigm.^53 54^ It is hypothesised, based on previous literature,^31 33 55–57^ that those who go on to develop delirium will display increased EEG slowing and attenuated ERP amplitudes as compared with those who do not. How this pattern varies as a function of subtype, severity and duration is an exploratory aim.

Characterising neural mechanisms of delirium vulnerability will lead to significant theoretical advances in the field of delirium neurophysiology. Furthermore, findings could feed into a delirium risk tool using EEG to identify individuals at high risk prior to surgery, a time during which preventative efforts can be employed.^58 59^ Such a tool could differentiate between risk of different subtypes, which have different care pathways and prognoses.^12 13^ Prevention of delirium is more effective than treatment once the delirium has occurred,^59^ and a recent meta-analysis reported that non-pharmacological multicomponent interventions reduced the incidence of delirium (risk ratio: 0.53; 95% CI 0.41 to 0.69).^60^

## Methods and analysis

### Study design

The study employs a prospective observational design to characterise associations between preoperative EEG power during resting states (eyes open and eyes closed), ERP components elicited during an auditory oddball paradigm, and delirium presence. Delirium subtype, severity, and duration will be explored as secondary outcomes. Cognitive status will be prospectively assessed. Delirium will be measured daily in hospital postoperatively and at discharge. This study is nested within two clinical trials, both of which are published.^61 62^

### Patient and public involvement

Neither the general public nor the patients were directly involved in the development or design of this study; however, clinical experts (geriatricians and interventional cardiologists) were involved in the study design.

### Outcomes

#### Primary outcome measures

The primary outcome of the current study is delirium: presence versus absence (as has been traditionally employed). We will compare differences in preoperative frequency band power, aperiodic offset and slope, and ERP waveforms between the groups. Cognitive status at baseline indexed by the Addenbrooke’s Cognitive Examination III (ACE-III) will be modelled as a covariate, along with age. Baseline cognitive impairment and age are major risk factors for incident delirium, carrying moderate to large effect sizes,^36^ and we want to identify functional brain associations of incident delirium independent of these risk factors.

#### Secondary outcome measures

The subtype (hypoactive, hyperactive and mixed), severity and duration of the delirium episode(s) will be explored as secondary outcomes. Group differences in preoperative frequency band power, aperiodic offset and slope, and ERP componentry will be assessed.

### Setting

This study is ongoing and conducted at multiple sites in Adelaide, South Australia. Recruitment and delirium assessments are conducted at the Royal Adelaide Hospital, where four specific sites are used: outpatient departments, intensive care, cardiothoracic and cardiology units. We collect data at participants’ homes, but offer the choice to come to the University of South Australia Magill campus if more convenient.

### Participants

Coronary artery bypass grafting (CABG) and transcatheter aortic valve implantation (TAVI) are both examples of precipitants after which delirium may manifest, with delirium occurring in approximately 25% of patients.^2 3^ The current study is recruiting older adults who can undertake assessments and are scheduled for elective CABG or TAVI at the Royal Adelaide Hospital. Participants for the present study are taken from two larger clinical trials. Inclusion and exclusion criteria for the studies are displayed in table 1. We chose these elective cohorts for practical reasons as the study has clinical relationships with these groups and it was not feasible to recruit from other surgical cohorts. There are no theoretical reasons why other elective surgical cohorts would produce different results.

**Table 1:** Clinical trial registration and inclusion and exclusion criteria for the two larger clinical trials in which participants for this study are recruited

	Clinical trial registration	Inclusion criteria	Exclusion criteria
CABG	Reducing delirium and dementia risk: a cognitive training intervention of older adults undergoing elective CABG surgery (clinical trial number: ACTRN12618000799257)	Male or female undergoing elective CABG at the Royal Adelaide Hospital Aged over 65 years Proficient in English Normal to corrected vision and hearing Live within 1-hour drive of Metropolitan Adelaide	Known learning disability Diagnosed dementia Diagnosed neurological or psychiatric disorder History of pharmaceutical cancer treatment (excluding purely surgical treatment) Stroke within the past year
TAVI	Development of risk models for cognitive decline and delirium in patients undergoing TAVI (clinical trial number: ACTRN12618001114235)	Male or female undergoing elective TAVI at the Royal Adelaide Hospital Aged over 60 years Participants with a clinical diagnosis of a neurodegenerative condition (including dementia) can be included	Current or recent (within the past year) alcohol or substance abuse or dependence Use of recreational drugs (within the past month) Diagnosed learning disability Insufficient English language, hearing (with aids) or vision (with glasses) to complete assessment tasks

CABG, coronary artery bypass grafting; TAVI, transcatheter aortic valve implantation.

### Sample size

With two groups (no delirium and delirium as an outcome), two covariates (age and cognitive status), the association with EEG/ERP predictors having a medium effect size (f: 0.30), α of 0.05 and power of 0.80, G*Power statistical analysis software estimates that a priori sample size of 90 participants is required.^63^ Notably, this power analysis does not account for shared variance across electrodes (see analytical approach for plan relevant to EEG and ERP data).

### Recruitment

Recruitment for this study is currently underway. Due to the global SARS-CoV-2 (COVID-19) pandemic, recruitment was halted twice in 2020 (March–July and November–December). Recruitment will continue until December 2022. Participants are recruited and complete data collection at least 1 week before their elective procedure. Potentially eligible participants identified through hospital databases are contacted via telephone or are seen in person at their preoperative clinic appointment. If eligible and willing to participate, they are scheduled for a data collection session to conduct the informed consent process.

### Procedure

**Figure 1:**
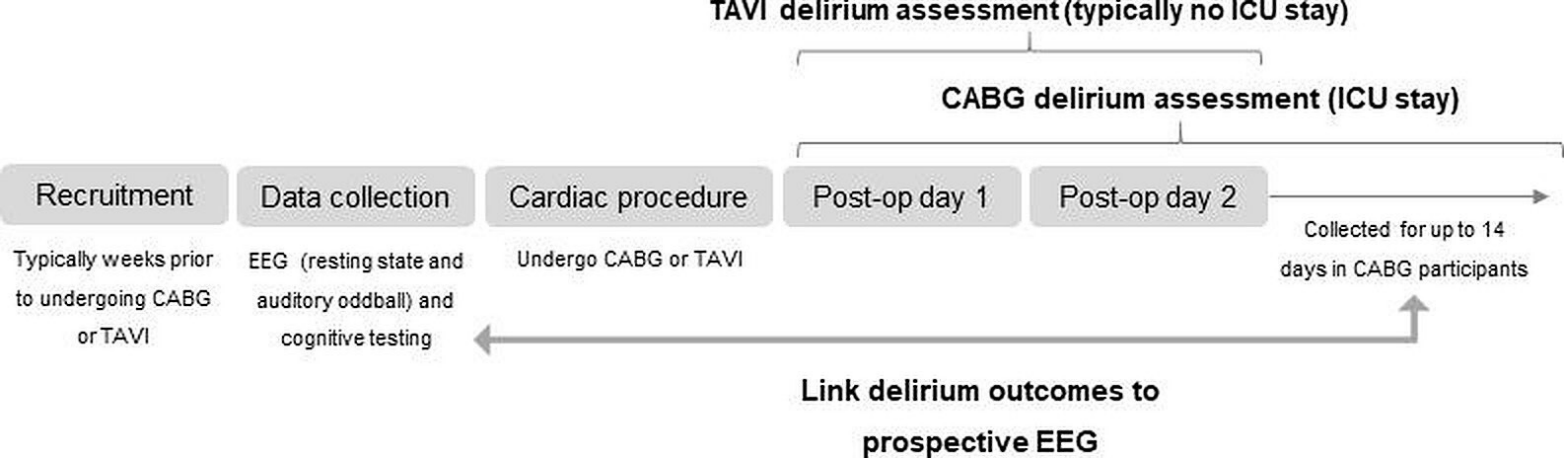
Study design. CABG, coronary artery bypass grafting; EEG, electroencephalography; ICU, intensive care unit; Post-op, postoperative; TAVI, transcatheter aortic valve implantation.

Approximately 1 or 2 weeks before their CABG or TAVI, data collection is conducted in participants’ homes or at the University of South Australia Magill campus if more convenient, and includes cognitive testing and EEG recording. Delirium is assessed daily postoperatively until discharge (in the case of TAVI patients discharged after 1 day, home delirium assessment is carried out on day 2). Within 7 days of discharge, an identical delirium assessment is conducted at the participants’ home. See figure 1 for an overview of the study design. Group allocation will be done retrospectively, with preoperative EEG data grouped according to postoperative delirium status (presence, severity, duration and subtype). Participants who develop delirium will form the ‘delirium group’ (hyperactive, hypoactive and mixed), and participants who do not develop delirium will form the ‘no delirium group’.

### Honoraria

Participants are remunerated for their time at the baseline data collection session with a $20 gift card.

### Measures

#### Cognitive function

Cognitive status is assessed before the procedure using the ACE-III, a global measure of cognitive function commonly used to screen for dementia.^64^ The ACE-III comprises five different cognitive domains, including attention, memory, language, verbal fluency and visuospatial ability, with a maximum score of 100. Higher scores indicate better cognitive function; specific subtotal scores include 18 points for attention, 26 points for memory, 14 points for verbal fluency, 26 points for language and 16 points for visuospatial ability.^64^ Cut-offs for dementia and mild cognitive impairment are characterised by scores lower than 82 and 88, respectively, and show high sensitivity (93%–100%) and specificity (96%–100%).^65 66^

#### Delirium assessment

Delirium presence, subtype, and severity will be captured using a comprehensive battery. To assess delirium in the intensive care unit, the Confusion Assessment Method (CAM) for the Intensive Care Unit (CAM-ICU) flowsheet will be used. The CAM-ICU features the four Diagnostic and Statistical Manual of Mental Disorders (DSM-III-R) delirium criteria: acute onset or fluctuating course, inattention, altered level of consciousness and disorganised thinking.^67^ It has high sensitivity (88% and 92%), specificity (92% and 100%) and interrater reliability (kappa.96), and is quickly administered.^67^ To assess delirium severity in ICU, the CAM-ICU 7 (CAM-ICU-7) will be used. The CAM-ICU-7 is a 7-point scale derived from CAM-ICU and Richmond Agitation Sedation Scale responses, and encompasses high internal consistency (Cronbach α: 0.85), and correlates well to the Delirium Rating Scale, Revised-98 (correlation coefficient: 0.64).^68^

Delirium assessment on the surgical wards will consist of the Memorial Delirium Assessment Scale (MDAS), which contains 10 items assessing: reduced level of consciousness, disorientation, short-term memory impairment, impaired digit span, reduced ability to maintain and shift attention, disorganised thinking, perceptual disturbance, delusions, decreased or increased psychomotor activity and sleep–wake cycle^69^. To help assess disturbances in arousal, the observational scale level of arousal (OSLA) is included in the ward assessments. The OSLA holds high sensitivity (0.87) and specificity (0.81), and characterises disturbances in arousal associated with delirium using four features: (1) eye opening, (2) eye contact, (3) posture and (4) movement.^70 71^ The MDAS (and OSLA) will ascertain a score of delirium severity and inform DSM-IV delirium presence or absence. The MDAS has high sensitivity (100%), specificity (95%), interrater reliability (κ: 0.92) and internal consistency (Cronbach α: 0.89).^72^ The short CAM is also collected, informed by MDAS and OSLA.

This comprehensive assessment will provide a dichotomous outcome for delirium (present/absent) along with the subtype, severity and duration of the delirium episode. Delirium will not be assessed by study staff on weekends. In the case of patients in hospital over the weekend, a chart-based review tool adapted from Inouye and colleagues will be used (74% sensitivity, 83% specificity and 0.41 interrater reliability κ)^73^. This method is not as extensive as the daily assessments during the working week, and may underestimate or inaccurately determine the presence of delirium.

#### EEG acquisition

We employ a 9-minute EEG recording using a 32-channel Ag/AgCI live electrode montage (Fp1, Fz, F3, F7, FT9, FC5, FC1, C3, T7, TP9, CP5, CP1, Pz, P3, P7, O1, Oz, O2, P4, P8, TP10, CP6, CP2, Cz, C4, T8, FT10, FC6, FC2, F8 and Fp2) positioned in an elastic cap according to the 10–10 system using Modified Combinatorial Nomenclature. EEG data is recorded using BrainVision Recorder (V.1.22.0001, Brain Products GmbH, Gilching, Germany) software at a sample rate of 1000 Hz, and is amplified by a LiveAmp amplifier (Brain Products GmbH, Gilching, Germany). We use actiCAPs (Brain Products GmbH, Gilching, Germany) with recording reference FCz, and ground Fpz electrode positions. Scalp electrode impedance will be kept below 10 kΩ before recording begins and if impedances drift above 25 kΩ during the recording, they will be interpolated.

The first 4 min of the recording constitutes the resting state period, comprised of 2 min eyes open and 2 min eyes closed. Immediately after, using Sennheiser Urbanite XL headphones, a 5-minute passive auditory oddball paradigm is employed, consisting of 300 stimuli of 150-millisecond stimulus duration and a 500-millisecond interstimulus interval; standard tones are presented at 600 Hz and deviant tones (23% of stimuli) at 1000 Hz. Sound density is set to −6 dBFS and the volume setting on the device is set to 86%. Participants are seated comfortably in front of a laptop placed on a table or available flat surface in the participant’s home. During the eyes open component of the resting state recording, participants are directed to look at a fixation point indicated by a cross in the centre of the laptop screen. During the oddball paradigm, participants are directed to watch a silent video on an iPad of passing traffic on a main road next to the University campus. Due to this being a clinically relevant protocol, we are unable to set individual auditory oddball parameters for participants, but we do check that they can hear the tones. Participants are shown the raw EEG signal to demonstrate common artefacts. They are instructed to relax, sit with their feet flat on the floor, and to avoid movement and excessive blinking.

### Data processing and analysis

Age and baseline cognitive function (two primary risk factors for delirium)^36^ will be used as covariates in our models. This will ensure that our EEG and ERP measures capture brain vulnerability to delirium independent of brain functional changes due to age and cognitive impairment. We will run sensitivity analyses covarying for procedure type (or stratified analyses if our numbers are too unbalanced), to ensure associations are not being driven by one patient group (CABG or TAVI). The EEG analysis approach combines measures of well-defined ERP components (eg, the MMN) and frequency bands (eg, theta) with data-driven approaches based on mass univariate analyses. Traditionally, most research has investigated the oscillatory (periodic) component of the EEG power spectra, but not the aperiodic background 1/f like component in which these oscillations are embedded.^74^ This aperiodic component has been found to change with ageing and cognitive state^75 76^ and contains features independent of oscillatory activity that appear to be physiologically relevant; failing to consider this aperiodic component may disguise physiologically relevant data.^54^ We will calculate traditional bandwith measures of the resting state data and will make these available.

### Power preprocessing

Resting state EEG data will be preprocessed in MATLAB (V.R2019a, The Mathworks, USA) using the EEGLAB toolbox V.v2019.1.^77^ We will remove bad or unused channels, and the data will be band-pass filtered from 1 Hz to 45 Hz. The data will be downsampled to 500 Hz and re-referenced to electrodes TP9 and TP10 before independent components analysis (ICA).^78^ ICLabel, an automated component classification method,^79^ will be applied to correct for ocular and muscle artefacts using an 80% threshold. Components identified as an 80% match to a previously identified artefact, that is, eye or muscle, will be removed.^79^ Bad or unused channels will be interpolated using clean data. Older adults have been shown to display a slower alpha frequency^80^ with alpha peaks around 8 Hz in some participants.^81^ Accordingly, in line with Tanabe and colleagues,^33^ we will set the lower limit of the alpha band to 6 Hz. From the aperiodic component, 1/f slope and offset features of the resting state EEG will be extracted using the open-source Python FOOOF (fitting oscillations and one over f) toolbox^74^ for comparison between groups. The FOOOF toolbox is available at https://githubcom/fooof-tools/fooof.

### ERP preprocessing

We will process ERP data in MATLAB V.R2019b with the ERPlab V.7.0.0 extension.^82^ Data will be re-referenced to electrodes TP9 and TP10. A 0.1 Hz high-pass filter, a 40 Hz low-pass filter and a 50 Hz notch filter will be applied and ICA will be performed on the filtered datasets. ICLabel will be used to remove bad components at a threshold of 80%. Bad channels will be interpolated using clean data. The data will then be low pass filtered at 20 Hz. Data will be epoched from −100 ms to +400 ms relative to auditory tone onset, and epochs containing amplitudes larger than ±100 μV will be excluded.

## Analytical approach

Averaged ERP data will be converted into three-dimensional spatiotemporal images for each participant and modelled using a mass-univariate general linear model implemented in statistical parametric mapping (SPM); the software is freely available.^83^ Statistical maps will be thresholded using family-wise error rate correction for multiple comparisons at a level of p <0.05, and clusters above the defined threshold will be examined. A cluster forming threshold of p <0.001 (uncorrected) will be used when the former (more conservative) approach does not reach significance. Only clusters p <0.05 cluster-corrected will be reported. This method allows for investigation of statistical effects across the entire dataset instead of using a priori time windows. Open-source MATLAB toolboxes Porthole and Stormcloud^53^ will be employed to visualise the scalp-time images created with SPM. Porthole and Stormcloud is available at https://githubcom/JeremyATaylor/Porthole.

We will also conduct traditional ERP analyses of the MMN and P3 component amplitude and latency to confirm our paradigm. We expect that components will display the typical age-related delays, where the MMN is found between 200 ms and 300 ms, and the P3 between 300 ms and 400 ms. Early components, which contribute to the MMN, including the P1 and N1, will be assessed in an exploratory manner. Standard analysis of covariance (ANCOVA) approaches will be used for the resting EEG and ERP component amplitude data. The independent variable will be delirium post procedure (delirium or no delirium) and the dependent variables will be the ERP amplitude and latency (MMN and P3), aperiodic offset and slope, and spectral power in theta, delta, alpha, beta and gamma frequency bands. Cognitive status (indexed by the ACE-III) and age will be modelled as covariates. We will categorise our electrodes into three regions: frontal, central and posterior, and divide our alpha by three (0.05/3) for the resting state EEG data. Independent samples t-tests will be used to assess differences across subtypes (hyperactive, hypoactive and mixed).

### Informed consent

This study is nested within two larger clinical trials, both of which have been approved for registration. Written consent is obtained from willing and eligible participants by study staff. Participants are reassured that participation is completely voluntary and that they can withdraw at any time, and that it will not affect the care provided to them while in hospital for their procedure.

### Data management

All identifying information are kept on a secure database (REDCap) accessible only by central study staff via institutional log in with individualised usernames and passwords. Information collected at recruitment is taken to secure storage in the laboratory immediately after.

### Risks

The study does not interfere with participants’ surgery or recovery and thus poses no additional risk to participants. EEG is completely non-invasive and poses no risk to participants. A small risk is posed to study staff when collecting data in participants’ homes, but this is mitigated by the use of a log-in safety application (HikerAlert), where if study staff fail to check in after a set amount of time, a nominated contact will receive an emergency message with their location.

### Ethical considerations

After cognitive testing, if a participant is found to score within the cut-offs for suspected dementia or mild cognitive impairment, results will be sent to their general practitioner for follow-up with the participants’ written consent (gained at baseline). While in hospital, if a participant is discovered to have delirium, study staff will alert a nurse or doctor to the assessment findings.

### Dissemination

It is anticipated that the results of this study will inform multiple publications, and will be presented at national and international conferences.
